# Abnormal Contingent Negative Variation Drifts During Facial Expression Judgment in Schizophrenia Patients

**DOI:** 10.3389/fnhum.2020.00274

**Published:** 2020-07-14

**Authors:** Qian Wang, Shenglin She, Lu Luo, Haijing Li, Yuping Ning, Jianjuan Ren, Zhangying Wu, Rongcheng Huang, Yingjun Zheng

**Affiliations:** ^1^Department of Clinical Psychology, Sanbo Brain Hospital, Capital Medical University, Beijing, China; ^2^Department of General Psychiatry, The Affiliated Brain Hospital of Guangzhou Medical University (Guangzhou Huiai Hospital), Guangzhou, China; ^3^School of Psychological and Cognitive Sciences, Beijing Key Laboratory of Behavior and Mental Health, Peking University, Beijing, China

**Keywords:** schizophrenia, contingent negative variation (CNV), N170, facial expression, personal and social performance (PSP)

## Abstract

Schizophrenia patients often show impaired facial expression recognition, which leads to difficulties in adaptation to daily life. However, it remains unclear whether the deficit is at the perceptual or higher cognitive level of facial emotion processing. Recent studies have shown that earlier face-evoked event-related potential (ERP) components such as N170 and P100 can effectively distinguish schizophrenia patients from healthy controls; however, findings for later waveforms are ambiguous. To clarify this point, in this study we compared electroencephalographic signals in schizophrenia patients and control subjects during a facial expression judgment task. We found that group effects of the occipital N170 and frontal lobe contingent negative variation (CNV) were both significant. The effect sizes (ESs) of N170 and CNV amplitudes were generally medium or small, whereas that of CNV slope for an upright face was large (>0.8). Moreover, N170 amplitude and CNV slope but not CNV amplitude was correlated with Personal and Social Performance (PSP) Scale score. These results suggest that the slope of CNV drift during facial expression processing has a potential clinical value for schizophrenia.

## Introduction

Schizophrenia is a psychiatric disorder characterized by social cognition deficits at the behavioral and neural levels (Onitsuka et al., 2013; Savla et al., 2013). One of the most prominent aspects of social cognition is the recognition of different facial expressions (Kohler et al., 2009; Savla et al., 2013). The neural mechanisms underlying this process have been investigated by neuroimaging and electroencephalography (EEG; Kohler et al., 2009; McCleery et al., 2015; Earls et al., 2016). Impaired social cognition in schizophrenia patients includes a failure to perceive social cues and the inability to perceive or share emotional experiences (reviewed by Green et al., 2015). Most EEG studies to date have focused on early-stage (P100 and N170) deficits in schizophrenia (Herrmann et al., 2004; Onitsuka et al., 2006; Tsunoda et al., 2012; Maher et al., 2016; Zheng et al., 2016). However, neuroimaging data suggest that in addition to the visual system, higher-order brain regions such as the prefrontal cortex and anterior cingulate cortex are abnormally activated in schizophrenia patients (Li et al., 2010; Taylor et al., 2012; Delvecchio et al., 2013), suggesting that there is a late event-related potential (ERP) component that distinguishes these individuals from healthy subjects.

To investigate this possibility, previous face processing ERP studies in schizophrenia focused on the group effects of N170 and N250 (Streit et al., 2001; Turetsky et al., 2007; Wynn et al., 2008, 2013; Lee et al., 2010; Jung et al., 2012). The occipitotemporal N170 waveform is observed in response to face stimuli, which showed lower amplitude and longer latency in schizophrenia patients (Zheng et al., 2016). The frontal-central N250, which could be modulated by the facial expressions, familiarity, and repetitions, also showed lower amplitude in schizophrenia patients (Streit et al., 2001; Wynn et al., 2008, 2013). A recent meta-analysis study systematically compared the effect sizes (ESs) of the N170 and N250 between schizophrenia patients and healthy controls and showed no significant difference (McCleery et al., 2015).

Contingent negative variation (CNV) is a slow negative potential that is presumed to reflect prefrontal functioning in top-down modulation (Walter et al., 1964). CNV deficits (decreased amplitude) preceding motor response has been reported in schizophrenia patients (Klein et al., 2000) and is thought to be associated with the frontal lobe hypometabolism observed in regional cerebral blood flow studies during the execution of tasks sensitive to frontal lobe dysfunction (Paulman et al., 1990; Frith et al., 1991; Andreasen et al., 1992). A recent study revealed that the emotional face evoked CNV amplitudes were lower in schizophrenia patients (Zhang et al., 2016). However, whether facial expression-related CNV could act as a better ERP index in distinguishing schizophrenia patients from healthy controls.

Schizophrenic patients also showed difficulty in processing face holistically (Shin et al., 2008; Kim et al., 2010; Bauser et al., 2012; Megreya, 2016). That defects could be measured by the face inversion effect (FIE), which is observed as a reduction in face discrimination performance for inverted faces compared to upright faces (Yin, 1969, 1970; Bauser et al., 2012). Previous studies suggested that the FIE of N170 is significantly reduced in people with schizophrenia (Tsunoda et al., 2012; Zheng et al., 2016). However, whether the FIE effect of late ERP components such as CNV exits defects in schizophrenic patients is still open to be explored.

Clinical symptoms of schizophrenia include positive symptoms such as hallucination and delusions and negative symptoms such as apathy and avolition; these are evaluated with the Positive and Negative Syndrome Scale (PANSS). There is increasing awareness that quality of life is important for individuals with mental illness. The Personal and Social Performance (PSP) scale—which measures social functioning in socially useful activities, personal and social relations, self-care, and disturbing and aggressive behavior—was developed based on the Social and Occupational Functioning Assessment Scale (Goldman et al., 1992) and is highly reliable for evaluating patients with schizophrenia. However, few studies have investigated the relationship between electrophysiological indices of facial expression processing and PSP scores.

This was addressed in the current study by EEG recordings using a 3 (facial expression: neutral/happy/sad) × 2 (face orientation: upright/inverted) design to simultaneously compare the group effects of N170 and CNV during an expression judgment task in schizophrenia patients and healthy control subjects. ESs and their correlations with PSP scale scores were calculated to determine whether ERP components are useful for schizophrenia evaluation.

## Materials and Methods

### Participants

A total of 20 patients diagnosed with schizophrenia according to Diagnostic and Statistical Manual of Mental Disorders, 4th Edition (10 females, mean age: 34.0 ± 7.2 years old) and 20 age-matched healthy control subjects (10 females, mean age: 32.4 ± 8.4 years old) were recruited for this study. All of the participants had a normal or corrected-to-normal vision and no history of medical or neurological disorders. Psychiatric symptoms were evaluated by a trained psychiatrist or psychologist according to the PANSS (Kay et al., 1987). The PSP scale was used to assess participants’ social functioning. Basic information on the study population is shown in Table 1.

**Table 1:** Clinical and demographic characteristics of schizophrenia patients and healthy control subjects.

	Schizophrenia patients (*n* = 20)	Healthy control subjects (*n* = 20)
Sex (male/female)	10/10	10/10
Education (years)	12.7 (2.6)	12.5 (3.7)
Handedness (right/left)	20/0	20/0
Schizophrenia subtypes		
Paranoid/undifferentiated	10/10	N/A
Duration of illness (years)	8.3 (7.0)	N/A
PANSS total	57.1 (16.1)	32.3 (1.5)
PANSS positive symptoms	15.6 (6.5)	7.4 (0.7)
PANSS negative symptoms	11.7 (4.6)	7.2 (0.4)
PANSS general symptoms	30.4 (8.1)	17.7 (1.2)
Antipsychotic medication	18/2	N/A
(Atypical/typical)

There was no history of any major psychiatric disorders or physical illnesses or use of medication known to affect the central nervous system among healthy controls. The exclusion criteria for both groups were obvious abnormalities by magnetic resonance imaging (MRI), neurological illness, traumatic brain injury, and substance use or addiction. The study protocol was approved by the Institutional Review Board of Guangzhou Brain Hospital. All participants provided written, informed consent, and were remunerated for their participation.

### Stimuli and Procedure

**Figure 1:**
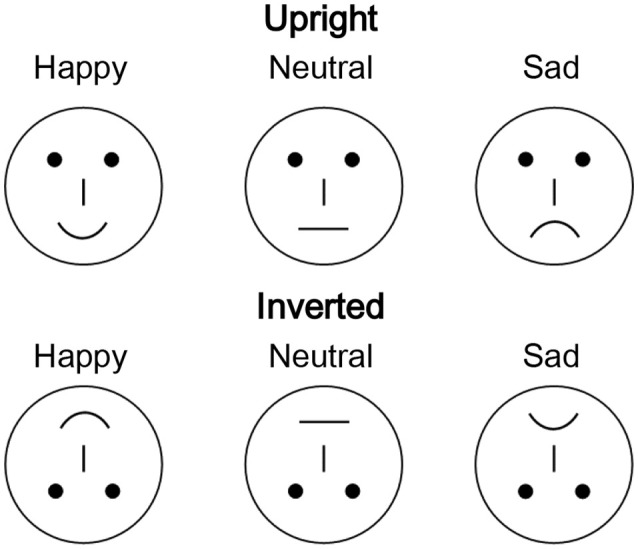
Examples of schematic face stimuli consisting of three facial expressions (happy, neutral, and sad) in the upright and inverted orientations.

In total, 54 different schematic faces with a neutral, sad, or happy expression were used as visual stimuli, with 18 individual schematic faces included for each stimulus type (Figure 1; She et al., 2017). Thus, each stimulus type included 18 models that were varied by altering the distance between the shapes of the facial features. The stimuli were presented at the fixation point with a visual angle of 7.27° × 6.06°.

During the EEG recording, participants were seated in a dimly lit room in a comfortable chair and were instructed to classify the facial expressions that were presented on the screen as neutral, happy, or sad, and to respond by pressing one of three different keys on the keyboard. The labels on the response buttons were balanced across participants. Each face was presented for 300 ms, with an inter-trial interval ranging randomly from 600 to 800 ms after the response. All 324 faces (three facial expressions, 108 faces for each) were randomly presented in three blocks. The participants first completed a practice sequence of 18 stimuli (six for each expression) that were not included in the analysis. Response accuracy and reaction time (RT) were recorded.

### Electrophysiological Recordings

Continuous EEG recording was performed with a set of 16 Ag/AgCl electrodes placed according to the 10/20 system. The EEG recording sites were F3, Fz, F4, C3, Cz, C4, P7, P3, Pz, P4, P8, PO7, PO8, O1, Oz, and O2. Electrooculography (EOG) was performed *via* electrodes placed on the bilateral external canthi and left infraorbital and supraorbital areas to monitor eye movements and blinking. Both EEG and EOG were sampled at 1,000 Hz with a 0.1–100 Hz band-pass using a Neuroscan NuAmps digital amplifier system (Neuroscan Labs, El Paso, TX, USA). The tip of the nose was used as the reference during recording, and approximate zero references was determined offline according to the reference electrode standardization technique^1^ (Yao, 2001). Electrode impedance was maintained below 5 kΩ.

### Data Analysis

Electrophysiological data were pre-processed with the EEGLAB toolbox (Delorme and Makeig, 2004) in the MATLAB environment. Long-term EEG signals were first filtered with a band-pass filter (0.5–40 Hz) and then segmented into epochs from −200 to 1,400 ms around the onset. To avoid the influence of hand reaction on electromyography, the time window of interest was then limited to −100 to 500 ms and the baseline correction was performed in the time window of −100 to 0 ms. Epochs containing potentials greater than ±1 mV were rejected as artifacts; the remaining epochs were then averaged to obtain an ERP for each electrode node and then low-pass filtered at 15 Hz.

In the individual level, mean amplitudes for N170 (160–180 ms) and CNV (200–400 ms) components were calculated. To calculate the slope of CNV, the ERP waveforms from 200–400 ms were first linear fit using the *polyfit* function in MATLAB environment. For each condition, the slope of the best fit function was taken as the CNV slope.

Statistical analyses including analysis of variance (ANOVA), the *t*-test, and Pearson’s correlation analysis were carried out using IBM SPSS Statistics 22 (SPSS Inc., Chicago, IL, USA). ESs were calculated with parietal *η*^2^ and Cohen’s *d*. *P*-values were corrected with the Bonferroni adjustment to avoid multiple comparisons. The null hypothesis rejection level was set at 0.05.

## Results

### Behavioral Performance

Behavioral performance was assessed for response accuracy and RT by 2 (group: patient/control) × 3 (facial expression: neutral/happy/sad) × 2 (face orientation: upright/inverted) three-way mixed-design ANOVA.

**Figure 2:**
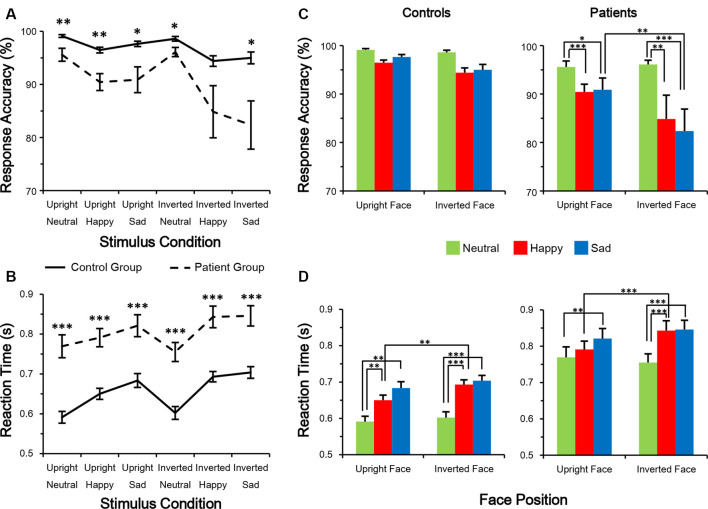
Performance in facial expression discrimination. **(A,B)** Response accuracy was higher **(A)** whereas reaction time (RT) was shorter **(B)** in control subjects than in schizophrenia patients. **(C,D)** Evaluation of differences among the six conditions. *p < 0.05; **p < 0.01; ***p < 0.001.

In the response accuracy analysis, only the main effect of group (*F*_(1,38)_ = 10.188, *p* = 0.003, partial *η*^2^ = 0.211) was significant. None of the other main and interaction effects were significant (all *p* > 0.05). A *post hoc* Bonferroni test showed no significance under facial expression conditions in the control group (Figure 2C). However, in the patient group, a happy or sad expression was detected less accurately than a neutral face (all corrected *p* < 0.05). The results of an independent samples *t*-test between groups (Figure 2A) showed that control subjects had higher response accuracy than patients (all *p* < 0.05, all Cohen’s *d* > 0.8), except under the inverted happy condition (*t*_(1,38)_ = 1.856, *p* = 0.071, Cohen’s *d* = 0.587).

In the RT analysis, the main effects of group (*F*_(1,38)_ = 29.131, *p* < 0.001, partial *η*^2^ = 0.434) and facial expression (*F*_(2,76)_ = 11.541, *p* < 0.001, partial *η*^2^ = 0.233) were significant. No other main or interaction effects were significant (all *p* > 0.05). A *post hoc* Bonferroni test showed that in both groups, RT was longer for detecting a happy or sad expression compared with a neutral expression (all corrected *p* < 0.01; Figure 2D). On the other hand, both groups had a shorter RT to detect a happy expression in an upright vs. an inverted face (both *p* < 0.01). The results of an independent-sample *t*-test between groups showed that control subjects had shorter RTs than patients (all *p* < 0.001, all Cohen’s *d* > 1.2; Figure 2B). These results indicate that compared with healthy control subjects, schizophrenia patients react more slowly and are more error-prone during the facial expression judgment task, suggesting an impairment in emotion processing.

### Main Effects of CNV and N170

**Figure 3:**
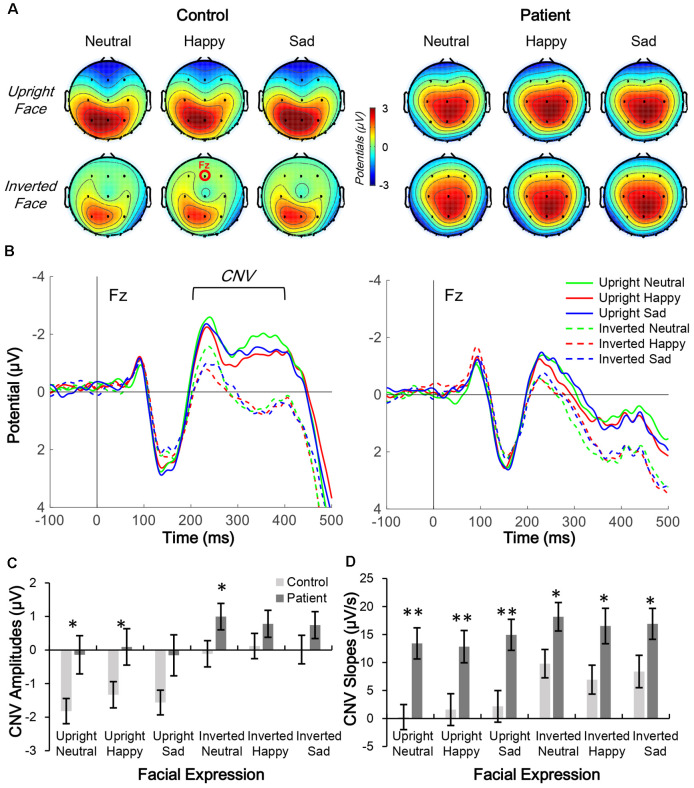
Comparison of contingent negative variation (CNV) topographic distribution in control and patient groups. **(A,B**) Topographic distribution of CNV mean amplitude (200–400 ms; **A**) and waveform of facial expression-evoked CNV (Fz electrode; **B**) under six conditions in control subjects (left panels) and schizophrenia patients (right panels). **(C,D)** Average CNV amplitude **(C)** and slope **(D)** under six conditions in the two groups, *p < 0.05; **p < 0.01.

**Figure 4:**
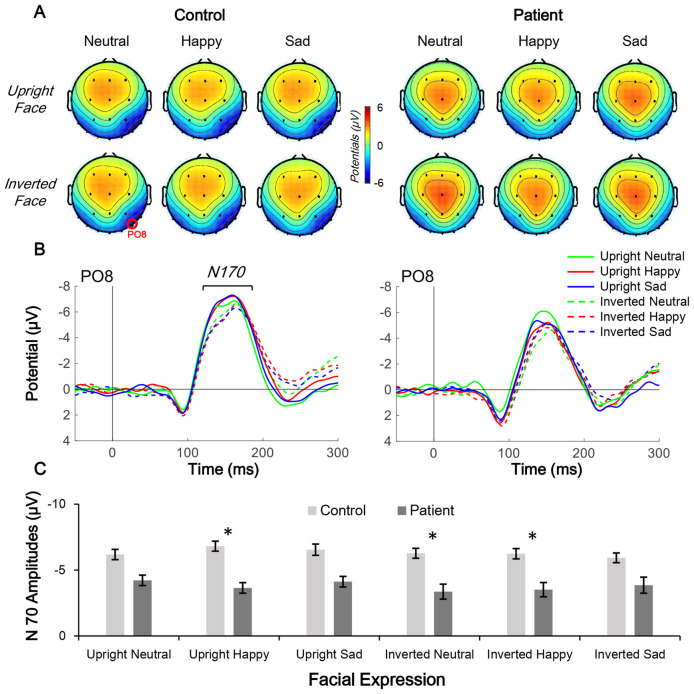
Comparison of N170 topographic distribution in schizophrenia patients and control subjects. **(A,B)** Topographic distribution of N170 mean amplitude (160–180 ms; **A**) and waveform of facial expression-evoked N170 (PO8 electrode; **B**) under six conditions in control subjects (left panels) and schizophrenia patients (right panels). **(C)** Average N170 amplitude under six conditions in the two groups, *p < 0.05.

The distributions of CNV (200–400 ms; Figure 3A) and N170 (160–180 ms; Figure 4A) were centered in the central-frontal and right temporo-occipital areas, respectively. Thus, electrodes Fz and PO8 were selected for three-way mixed-measure ANOVA for CNV and N170, respectively.

CNV waveforms in the frontal lobe differed under the six experimental conditions in each group (Figure 3B). Furthermore, the CNV waveform had a lower amplitude and steeper slope in patients than in controls.

In the CNV amplitude analysis, the main effects of group (*F*_(1,38)_ = 4.222, *p* = 0.047, partial *η*^2^ = 0.100), facial expression (*F*_(2,76)_ = 3.496, *p* = 0.035, partial *η*^2^ = 0.084), and inversion (*F*_(1,38)_ = 16.896, *p* < 0.001, partial *η*^2^ = 0.308) were all significant. No interaction effect was found (all *p* > 0.05). The results of the *post hoc* Bonferroni test showed that in the control group, the CNV evoked by an upright face was larger than those evoked by an inverted face (all corrected *p* < 0.001), whereas the same inversion effect was also found in the patient group (all corrected *p* < 0.05). Facial expression was non-significant in both groups.

In the CNV slope analysis, the main effects of group (*F*_(1,38)_ = 9.805, *p* = 0.003, partial *η*^2^ = 0.205) and inversion (*F*_(1,38)_ = 6.213, *p* = 0.017, partial *η*^2^ = 0.141) were significant, but the main effect of facial expression was not (*F*_(2,76)_ = 0.049, *p* = 0.953, partial *η*^2^ = 0.001). No interaction effect was found (all *p* > 0.05). The results of the *post hoc* Bonferroni test showed that in the control group, the slope of CNV evoked by an upright face was smaller than that evoked by inverted face for all three facial expressions (all corrected *p* < 0.001), whereas in the patient group, the inversion effect was only observed under the neutral condition (corrected *p* = 0.014) and not under the happy or sad condition (both corrected *p* > 0.1). No significance was found for facial expression in either group.

In the N170 amplitude analysis, only the main effect of group (*F*_(1,38)_ = 4.705, *p* = 0.036, partial *η*^2^ = 0.110) was significant; other main and interaction effects were non-significant (all *p* > 0.05). The results of the *post hoc* Bonferroni test found no significant effects (both corrected *p* > 0.05).

In summary, although the main effects of each group were significant for the three ERP indices, the ES of CNV slope was larger than those of CNV and N170 amplitude.

### ESs of CNV Amplitude and Slope and N170 Amplitude

**Figure 5:**
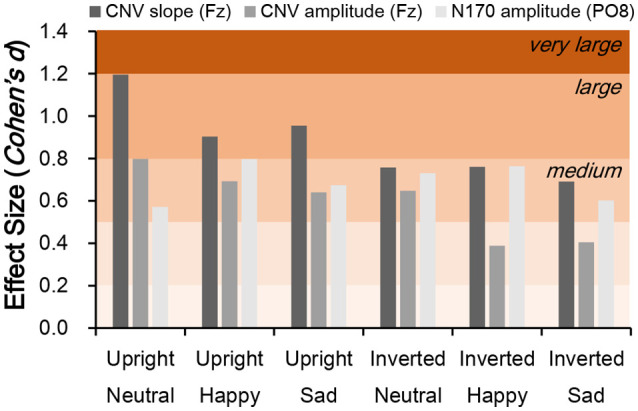
Comparison of effect size (ES; Cohen’s *d*) for CNV slope, CNV amplitude, and N170 amplitude between schizophrenia patients and control subjects. Cohen’s *d* > 0.2, small effect; *d* > 0.5, medium effect; *d* > 0.8, large effect.

Independent *t*-tests were performed for the three ERP indices to estimate the between-group difference under each condition. For CNV amplitude, inter-group difference was only significant under upright neutral (*t*_(38)_ = 2.522, *p* = 0.016, Cohen’s *d* = 0.789), upright happy (*t*_(38)_ = 2.191, *p* = 0.035, Cohen’s *d* = 0.693), and inverted neutral (*t*_(38)_ = 2.047, *p* = 0.048, Cohen’s *d* = 0.647) conditions with medium ESs (Figure 3C). For CNV slope, the difference was significant under upright neutral (*t*_(38)_ = 3.781, *p* = 0.001, Cohen’s *d* = 1.196), upright happy (*t*_(38)_ = 2.857, *p* = 0.007, Cohen’s *d* = 0.904), and upright sad (*t*_(38)_ = 3.020, *p* = 0.005, Cohen’s *d* = 0.955) conditions with large ESs and under inverted neutral (*t*_(38)_ = 2.397, *p* = 0.022, Cohen’s *d* = 0.758), inverted happy (*t*_(38)_ = 2.406, *p* = 0.021, Cohen’s *d* = 0.761), and inverted sad (*t*_(38)_ = 2.184, *p* = 0.035, Cohen’s *d* = 0.691) conditions with medium ESs (Figure 3D). For N170 amplitude, the inter-group difference was only significant under upright happy (*t*_(38)_ = 2.238, *p* = 0.031, Cohen’s *d* = 0.797), inverted neutral (*t*_(38)_ = 2.12, *p* = 0.041, Cohen’s *d* = 0.731), and inverted happy (*t*_(38)_ = 2.306, *p* = 0.027, Cohen’s *d* = 0.764) conditions with medium ESs (Figure 4). A comparison of ESs of the three ERP indices under each condition revealed that only the CNV slope under the upright face condition had a large ES (Figure 5).

### ERP Indices Correlated With Schizophrenia Symptoms and PSP Scores

Pearson correlations between ERP indices and positive symptoms, negative symptoms, and PSP scores were calculated. The N170 amplitude was positively correlated with positive symptoms (Table 2), whereas N170 amplitude and CNV slope but not CNV amplitude was negatively correlated with PSP score (all *p* < 0.05). These results indicate that both N170 amplitude and CNV slope predict social performance in schizophrenia patients and distinguishes them from healthy controls.

**Table 2:** Pearson correlation coefficients between event-related potential (ERP) indices and clinical evaluation scores.

		CNV slope		CNV amplitude		N170 amplitude	
		Upright face	Inverted face	Upright face	Inverted face	Upright face	Inverted face
Positive symptoms	*r*	0.201	0.310	0.174	0.029	0.415**	0.495**
	*p*	0.213	0.052	0.282	0.859	**0.008**	**0.001**
Negative symptoms	*r*	0.153	0.137	0.100	0.134	0.134	0.146
	*p*	0.345	0.398	0.539	0.410	0.410	0.369
PSP	*r*	−0.374*	−0.382*	−0.236	−0.162	−0.371*	−0.412**
	*p*	**0.017**	**0.015**	0.143	0.317	**0.019**	**0.008**

PANSS, Positive and Negative Syndrome Scale; PSP, Personal and Social Performance Scale. *p < 0.05; **p < 0.01. Bold fonts represent significant correlation coefficients.

### CNV Indices Correlated With Behavioral Indices

**Figure 6:**
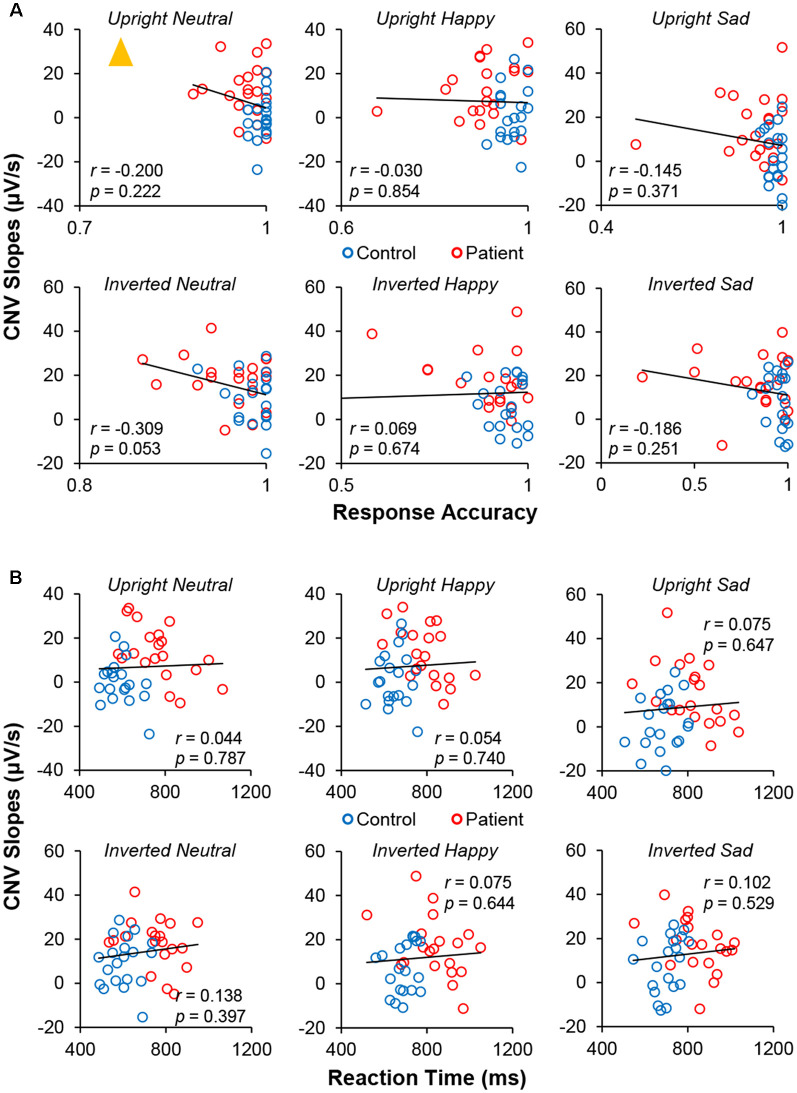
Pearson correlation measurements between CNV slopes and **(A)** response accuracy and **(B)** RT under six conditions. The *yellow triangle* represents the outlier point.

Pearson correlations between CNV indices (slope and amplitude) and behavioral indices (RT and accuracy) were calculated. As shown in Figure 6A, no significance was found between CNV slope and accuracy, which might be still limited by the accuracy variation. No significant correlation was found between CNV slopes and RTs (Figure 6B). No significance was found between CNV amplitude and behavioral indices (Table 3). Together, these results failed to reveal any meaningful relationship between CNV and behavioral performance.

**Table 3:** Pearson correlation coefficients between contingent negative variation (CNV) indices and behavioral indices.

		Upright neutral	Upright happy	Upright sad	Inverted neutral	Inverted happy	Inverted sad
CNV slope (Fz) vs. RT	*r*	0.044	0.054	0.075	0.138	0.075	0.102
	*p*	0.787	0.740	0.647	0.397	0.644	0.529
CNV slope (Fz) vs. Accuracy	*r*	−0.326*	−0.030	−0.145	−0.309	0.069	−0.186
	*p*	**0.040**	0.854	0.371	0.053	0.674	0.251
CNV amplitude (Fz) vs. RT	*r*	0.134	0.115	0.228	0.109	0.118	0.164
	*p*	0.410	0.478	0.157	0.504	0.468	0.312
CNV amplitude (Fz) vs. Accuracy	*r*	−0.199	−0.133	−0.232	−0.165	−0.268	−0.246
	*p*	0.219	0.415	0.149	0.309	0.095	0.127

RT: reaction time; *p < 0.05. Bold fonts represent significant correlation coefficients.

## Discussion

The dysfunction in affective face processing in schizophrenia patients has been widely explored in previous studies using behavioral and neurophysiological approaches (Kohler et al., 2009; Li et al., 2010; Taylor et al., 2012; Delvecchio et al., 2013; Savla et al., 2013; McCleery et al., 2015); however, only a few have simultaneously compared the perceptual and cognitive ERP components evoked by task-relevant facial expressions between schizophrenia patients and healthy subjects (reviewed by McCleery et al., 2015). In the current study, we measured early and late ERP components during a facial expression detection task and found the following: (1) schizophrenia patients exhibited abnormalities in early (N170) and late (CNV) facial expression processing; (2) N170 amplitude and CNV slope were correlated with social performance; and (3) only the CNV slope under the upright face condition had a large ES (>0.8).

Face perception is one of the most important social cues in daily life and is impaired in individuals with schizophrenia (Green et al., 2015). Following previous findings (Kohler et al., 2009; Savla et al., 2013), in the current study schizophrenia patients performed poorly in the identification of facial expressions, reacting more slowly and making more errors in the facial expression judgment task.

The neural correlates of schizophrenia-related deficits have been extensively studied by functional fMRI and ERP analysis (Li et al., 2010; Taylor et al., 2012; Delvecchio et al., 2013; McCleery et al., 2015) fMRI studies have revealed that facial expression processing is correlated not only with activation of association cortices but also with altered activation of high-order limbic-cortical circuits, frontal cortex, and putamen (Haxby et al., 2002; Vuilleumier and Pourtois, 2007; Sabatinelli et al., 2011). On the other hand, ERP studies with high temporal resolution have revealed consistent and significant impairment in both early (P100 and N170) and late (N250 and CNV) visual ERP amplitudes in schizophrenia (McCleery et al., 2015; Earls et al., 2016; Zhang et al., 2016). In the current study, significant group effects were observed not only for N170 amplitude but also for CNV amplitude and slope, suggesting aberrant hierarchical processing in individuals with schizophrenia. However, it is unclear whether there is greater impairment in the earlier or later stage of affective face processing. Meta-analyses have demonstrated that ES estimates reveal a smaller degree of impairment at the neural level (P100, *ES* = 0.41; N170, *ES* = 0.64; N250, *ES* = 0.49; McCleery et al., 2015; Earls et al., 2016) than that observed in emotion-based tasks (*ES* = 0.89; Savla et al., 2013). In the current study, we compared parietal *η*^2^ values of the group main effect in the ANOVA to estimate whether these can distinguish schizophrenia patients from healthy controls and determined that parietal *η*^2^ values of N170 and CNV amplitudes (both approximately 0.1) were lower than those of accuracy (parietal *η*^2^ = 0.211) and RT (parietal *η*^2^ = 0.434), whereas only the ES of the CNV slope was comparable to the behavioral indices (parietal *η*^2^ = 0.205). A recent ERP study of facial expression in schizophrenia showed that the parietal *η*^2^ of the group effect of CNV amplitude was markedly smaller than those of P100 and N170 amplitudes (Zhang et al., 2016); however, these investigators did not examine the slope the CNV evoked by facial affect.

Cohen’s *d* under each stimulation condition was further compared across N170 and CNV amplitudes and CNV slope. We found that Cohen’s *d* values of N170 and CNV amplitudes ranged from 0.389–0.798 (small to medium ESs), whereas that of CNV slope under the upright face condition were all larger than 0.8 (large ESs; Figure 5). On the other hand, the *d* value of the CNV slope under the inverted face condition ranged from 0.691–0.761. These results explain why the amplitude of the late ERP component has an ES similar to those of early components such as N170 (McCleery et al., 2015), and suggest that slow drifts in CNV are a characteristic of schizophrenia.

In this study, we examined N170 originating in the temporo-occipital lobe based on the CNV waveform detected by frontal lobe electrodes. Previous research has shown that schizophrenia patients exhibit biochemical, molecular, and physiological changes in the frontal lobe (Johnston-Wilson et al., 2000; Jaffe et al., 2016). Neuroimaging studies have revealed that in addition to abnormal or lack of activation in the frontal cortex during an emotion judgment task (Takahashi et al., 2004; Ferrarelli et al., 2008; Minzenberg et al., 2009; Ursu et al., 2011), a disconnect between the frontal and other cortices is observed in schizophrenia (Ellison-Wright and Bullmore, 2009; Bjorkquist et al., 2016).

Given that slow CNV drifts reflect low-frequency EEG components related to top-down processing (Chennu et al., 2013), the change in CNV slope suggests a deficit in the high-order cognitive processing in schizophrenia patients. As shown in Figure 5, the ESs of CNV slopes were generally larger under upright face conditions, rather than inverted face conditions. On the other hand, the behavioral performance was also correlated with CNV slopes were generally larger under upright face conditions, but not under inverted face conditions. Therefore, the deficits of CNV slopes might be the neural correlates which interpret the abnormal social communications in schizophrenia patients.

Limitations of this study were the small sample sizes and the absence of conditions without facial expression judgment, which made it difficult to distinguish whether the CNV slope is involved in emotion analysis, movement initiation, or executive control. Moreover, it remains unclear whether the observed change in CNV slope is specific to schizophrenia or is a general feature of mental disorders. This question will be addressed in future studies.

In conclusion, although meta-analyses of EEG studies of facial expression processing in schizophrenia have shown consistent results for early-stage components, the utility of late components for discriminating schizophrenia patients and healthy subjects is unknown. Our findings reveal that the slope of CNV originating in the frontal lobe has clinical value for individuals with schizophrenia, indicating that late ERP components warrant closer examination.

## Data Availability Statement

The datasets generated for this study are available on request to the corresponding author.

## Ethics Statement

The studies involving human participants were reviewed and approved by Institutional Review Board of Guangzhou Brain Hospital. The patients/participants provided their written informed consent to participate in this study.

## Author Contributions

QW, SS, LL, HL, YN, JR, ZW, RH, and YZ designed and wrote the protocol for the study. QW, SS, and HL performed the experiments. QW and LL analyzed the data and wrote the first draft of the manuscript. All authors contributed to and approved the final manuscript.

## Conflict of Interest

The authors declare that the research was conducted in the absence of any commercial or financial relationships that could be construed as a potential conflict of interest.

## Footnotes

^1^www.neuro.uestc.edu.cn/rest
